# A highly compact Fabry Perot cavity-based MIMO antenna with decorrelated fields

**DOI:** 10.1038/s41598-022-18050-w

**Published:** 2022-08-18

**Authors:** M. Umair Illahi, Muhammad U. Khan, Rifaqat Hussain, Farooq A. Tahir

**Affiliations:** 1 School of Electrical Engineering and Computer Science, National University of Sciences and Technology (NUST), Islamabad, Pakistan; 2 Department of Electrical Engineering, King Fahd University of Petroleum and Minerals (KFUPM), Dhahran, Saudi Arabia

**Keywords:** Engineering, Electrical and electronic engineering

## Abstract

This work presents the design of a low profile Fabry-Perot cavity-based multiple-input-multiple-output antenna with low correlation coefficient. The fields of closely placed antenna elements are tilted by using a phase-gradient partially reflective surface (PRS), thereby decreasing the correlation coefficient. The PRS is designed in such a way that its reflection phase is complementary to that of the ground plane. The PRS decorrelates the fields of the two radiating elements when placed at a height of *λ/*10 above them resulting in a reduction of the correlation coefficient by almost 95% for an isotropic environment. This height is considerably less than *λ/*4, which has been reported previously.

## Introduction

Ever growing numbers of active devices are putting more and more stress on effective management of the most valuable resource in wireless communication i.e., the wireless spectrum. Multiple-Input-Multiple-Output (MIMO) systems are being used ubiquitously in modern communication systems to meet the requirements of high data rate and reliable communication. Current 4G/LTE standards rely on MIMO systems to achieve high data rates while remaining within reasonable limits of bandwidth (BW) and transmitted power. To address the needs of future 5G communication standards, MIMO systems will continue to be of main focus. 5G is expected to operate in the mm-wave range for close proximity communication. At the same time, it will use the sub-6 GHz frequency bands for wider coverage and backward compatibility with 4G/LTE networks. All these frequency ranges have their own design challenges that are being addressed simultaneously in academia and in industry. Although multiple teams work in parallel to design a completely functioning wireless system, antenna design still plays a pivotal role in the process.

The performance of MIMO systems among other parameters, rely on the number of uncorrelated communication channels. Correlation among channels is a function of the radiation patterns of the MIMO antenna elements and the propagation environment. This correlation is described by a parameter called the correlation coefficient (*ρ*), which has a normalized value between 0 and 1, and which represent completely uncorrelated and correlated channels, respectively. To fully exploit the benefits of MIMO systems, completely uncorrelated channels are desired. The coefficient *ρ* is generally calculated using the complex 3-D radiation patterns of the antenna elements operating in an isotropic propagation environment^1^ with balanced polarization. This essentially leaves *ρ* as a function of antenna parameters only. It has, therefore, become an important parameter which is used to evaluate the performance of a MIMO antenna design.

Calculation of *ρ* using field-based equation is an easy assignment in a simulation software however practically it is a very tedious task as it requires complex 3D measurements of radiation patterns of individual antenna elements along with post processing of the measured data. Consequently, a computationally simple S-parameter-based expression has also been proposed for the calculation of *ρ*^2^. In this expression^3^ there is a direct correlation between reduction of *ρ* and port coupling among antenna elements. Active research has been carried out to develop techniques that increase the isolation in MIMO antennas. Some of the techniques include use of the decoupling networks^4^, neutralization lines^5^, negative group delay lines^6,7^, defected ground planes^**?**^ and metamaterials^8^. All such work reports the reduction of *ρ*, ostensibly through the increase in isolation among MIMO antenna elements^16^. However, recently it has been shown that calculating *ρ* using this expression, often leads to inaccurate results^9^. Consequently, actual correlation between the radiated fields of individual antenna element has to be reduced in order to decrease *ρ*. A technique has been proposed in^10^ to accomplish this by decorrelating the radiated fields of individual antenna elements. This decorrelation is achieved by placing a Partially Reflecting Surface (PRS) with phase gradient over a MIMO antenna at a height of *λ*/4. This height of proposed design is impractical for many of the compact wireless devices, especially in sub-6 GHz frequency bands.

To address the issue of structural height of the antenna, this work proposes a design suitable for compact wireless devices in sub-6 GHz frequency bands. To achieve this reduction of height while reducing *ρ*, a negatively phased PRS has been designed to be used with MIMO antenna. This PRS when placed at the reduced height of *λ*/10 over the MIMO antenna, essentially forms a Fabry Perot cavity. Resulting waves being radiated from this compact structure are decorrlated with reduced *ρ*. The reduced height of *λ*/10 of FP cavity corresponds to 5.6 mm at 5.36 GHz making it a suitable design for practical applications.

The rest of the paper is organized as follows. Section “Design methodology” describes the design methodology for the low-profile FP cavity-based MIMO antenna design. Section “Proposed design” details the proposed phase-gradient PRS with negative reflection phase as well as the complete MIMO antenna design. In Sect. “Results and discussion”, full-wave simulation and measured results of the prototype design are discussed in detail with main focus on reduction of *ρ*. Finally, some conclusions are presented in Sect. “Conclusion”.

## Design methodology

The objective of the proposed design is to reduce the height of an FP cavity resonator in MIMO antenna configurations and still achieve a reduced *ρ*. Note that *ρ* is a function of both the radiation patterns of the MIMO antenna elements and the propagation environment. However, in most cases, a fixed isotropic propagation environment with a balanced polarization is assumed when evaluating the performance of MIMO antennas. In such a scenario, *ρ* is only a function of the radiation patterns of the MIMO antenna elements. The expression for *ρ* in such a condition is given as^1^:

1 $$\rho = \frac{{\int {\int_{0}^{4\pi } {\left| {\left[ {{\varvec{F}}_{1} \left( {\theta , \varphi } \right)*{\varvec{F}}_{2} \left( {\theta , \varphi } \right)} \right]d\Omega } \right|} } }}{{\sqrt {\int {\int_{0}^{4\pi } {\left| {{\varvec{F}}_{1} \left( {\theta , \varphi } \right)} \right|^{2} d\Omega \int {\int_{0}^{4\pi } {\left| {{\varvec{F}}_{2} \left( {\theta , \varphi } \right)} \right|} } } }^{2} d\Omega } }}$$

where F_1_ and F_2_ represent the complex field patterns of elements 1 and 2 of the MIMO antenna, respectively. Eq. (1) shows that *ρ* can be reduced in three different ways. First of these is to manipulate the relative phase of the antenna elements such that the numerator becomes small. This is mainly achieved by spatially separating the antenna elements. Some isolation techniques also affect the relative phase and helps to reduce *ρ*. The second way to achieve a low *ρ* is to place the elements orthogonal to each other to render their polarization also orthogonal with respect to each other, and thereby reduce *ρ*. Lastly, the main beam directions of individual antenna elements can be so tilted relative to each other that the numerator in Eq. (1) becomes small, thereby, decreasing *ρ*.

The method of beam tilting to reduce *ρ* has been reported in^10^ for closely placed antenna elements. Radiated beams of each element are tilted away from each other thus reducing their spatial correlation. This beam tilt in each element is achieved by designing an FP cavity-based MIMO antenna wherein a phase-gradient PRS is used. This phase gradient PRS is placed at an appropriate height over the primary radiating antenna elements which essentially forms a resonance FP cavity. This technique has shown to reduce the *ρ* by almost 95%.

The design technique of^10^ requires the antenna to be in the FP cavity configuration, thus making the antenna non-planar. From^11^, the height of the FP cavity is given by:

2 $$h = \left( {\varphi_{{{\text{PRS}}}} + \varphi_{G} } \right)\frac{\lambda }{4\pi } + N\frac{\lambda }{2} , N \in { }Z$$

where *φ*_PRS_ is the reflection phase of the PRS; *φ*_*G*_ is the reflection phase of the ground plane; *λ* represents the free space wavelength and *N* represents the excitation mode of the FP cavity. Usually, the cavity is excited at the dominant mode for which *N* = 0. Therefore, the reflection phases *φ*_PRS_ and *φ*_*G*_ essentially dictate the choice for height of the cavity. In a standard FP cavity both surfaces are nearly Perfect Electric Conductors (PECs), essentially each providing a reflection phase of *π*. According to Eq. (2) this makes the height of cavity *λ*/2. Following the design approach presented in^10^, a 4-element MIMO antenna with PRS has been presented in^12^ while dielectric resonator-based MIMO antennas have been described in^13^ and^14^. In all of these designs, a cavity height of *λ*/4 was achieved by designing the phase-gradient PRS to be an Artificial Magnetic Conductor (AMC) which offers 0^0^ *φ*_*PRS*_ instead of *π*.

Theoretically, the height of the cavity can be tailored to any desired value by manipulating the reflection phases *φ*_PRS_ and *φ*_*G*_, albeit over a narrow band. Comprehensive research work has been carried out to investigate ways by which the height of the FP resonator can be reduced for a single antenna element. The techniques employed rely on altering the reflection phase *φ*_PRS_^15^, *φ*_*G*_^16^, or both, in a complementary fashion^17^ to design the ultrathin FP cavities. Technique of altering the *φ*_PRS_, requires it to present reflection phase closer to -*π* so it can complement the *φ*_*G*_ having *π* value. Similarly, *φ*_*G*_ can also be designed to present reflection phase to complement *φ*_PRS_. More closely these two surfaces complement each other in term of reflection phase, more compact cavities can be realized. Ultrathin cavities are formed by simultaneous tailoring of *φ*_*G*_ and *φ*_PRS_ so that they almost cancel each other at resonance frequency. Most of the previous works only attempt to change the *φ*_PRS_ because it does not affect the antenna operation. Once a PRS is placed at the correct height which satisfies the reflection phase condition in accordance with Eq. (2), a resonant FP cavity is formed from which waves escape in a coherent manner thus making radiated fields more directive^11^.

Antenna designs with tilted beams using the FP cavity-based configuration can also be found in the literature, as for instance in^18–20^, where phase-gradient PRSs are utilized. Such PRSs are made up of non-uniform unit cells which offer gradual decrease in the reflection coefficient magnitude in a particular axis, thus enabling the beam to tilt. To achieve higher degree of tilt, change between unit cells of maximum and minimum reflection unit cell has to be gradual which increases the lateral size of the structure. The reduced FP cavity height based work is also limited to a single radiating source.

The design proposed herein utilizes a phase-gradient PRS to tilt the beams of two radiating elements in a MIMO antenna in opposite directions to reduce *ρ*. However, it is designed to be placed at an ultra-low height for which the reflection phase of the PRS is altered. The PRS is so designed that its reflection phase approaches −*π*, which is the complement of the reflection phase of the ground plane. At the same time, an important design consideration is to maintain a low coupling between the closely placed antenna elements even as the PRS is introduced.

## Proposed design

### Unit cell design

For our proposed design, we have chosen a unit cell with capacitive square patches at the bottom side being illuminated by the source and an inductive grid on the top side. Such a structure exhibits partial reflection over wide range of the dimension of the capacitive square, and it also achieves a slow phase variation with frequency. This slow variation is necessary to keep the phase of the reflection coefficient of the unit cell negative while tapering its amplitude gradually to introduce the beam tilt. This decrease can be achieved by gradually changing the dimensions of the capacitive grid. Also, both lateral dimensions of the unit cell must remain below *λ*/2 to avoid grating lobes.

**Figure 1:**
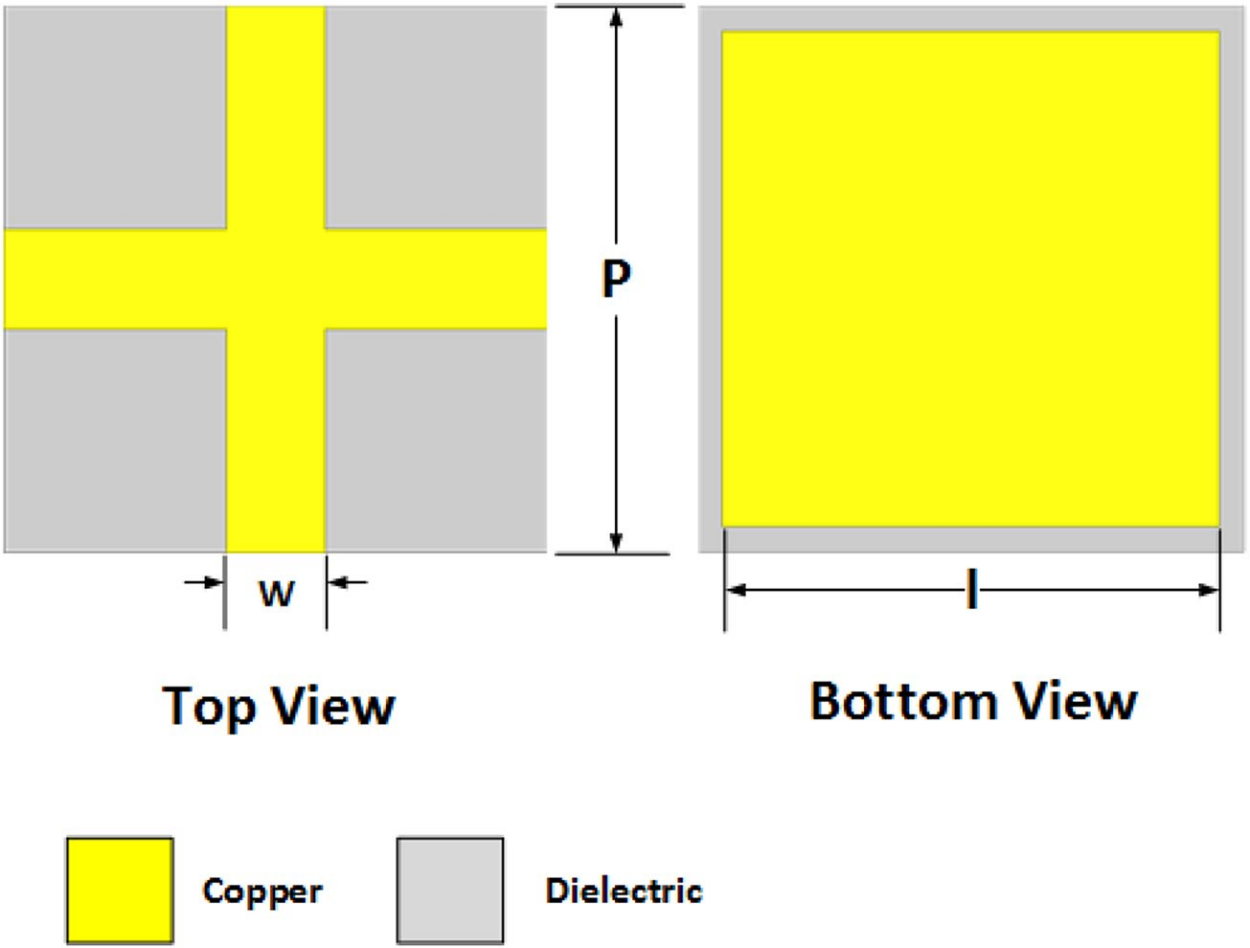
Unit cell of the proposed phase gradient PRS.

**Figure 2:**
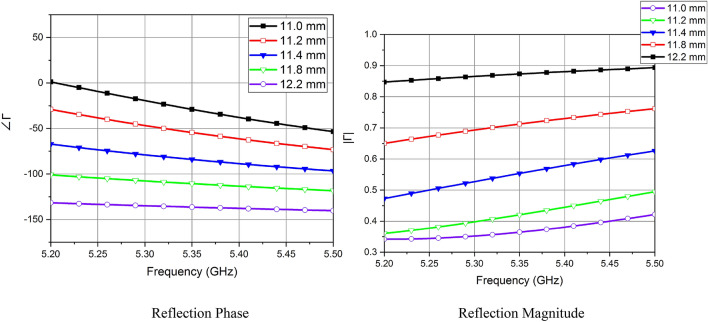
Unit Cell Design and its Results.

The unit cell is realized on a RO4003C laminate (*ε*_*r*_ =3.55) with a thickness of 0.813 mm and is modeled and analyzed by using the simulation software High-Frequency Structure Simulator (HFSS). Top and bottom views of the unit cell are shown in Fig. 1. The Inductive grid has a constant width *w* = 2 mm, while capacitive patches have width *l* varying from 12.2 mm to 11 mm, so that their specific arrangement can be used to construct a phase gradient surface. The periodicity of the unit cell *p* is kept at 13 mm. The magnitude and phase of the designed unit cell are shown in Fig. 2. A decrease in the reflection magnitude of the unit cell with a decrease in patch width is observed in the 5–5.5 GHz band, while the reflection phase remains negative. The magnitude and phase of the reflection coefficient at 5.36 GHz are tabulated in Table 1.

**Table 1:** Unit Cell Analysis at 5.36 GHz with fixed *w* = 2 mm and varying *l.*

Unit Cell	*l* (mm)	|Γ|	∠Γ
R1	12.2	0.87	− 136.70
R2	11.8	0.72	− 111.218
R3	11.4	0.56	− 84.98
R4	11.2	0.43	− 55.85
R5	11.0	0.37	− 30.79

### PRS design

**Figure 3:**
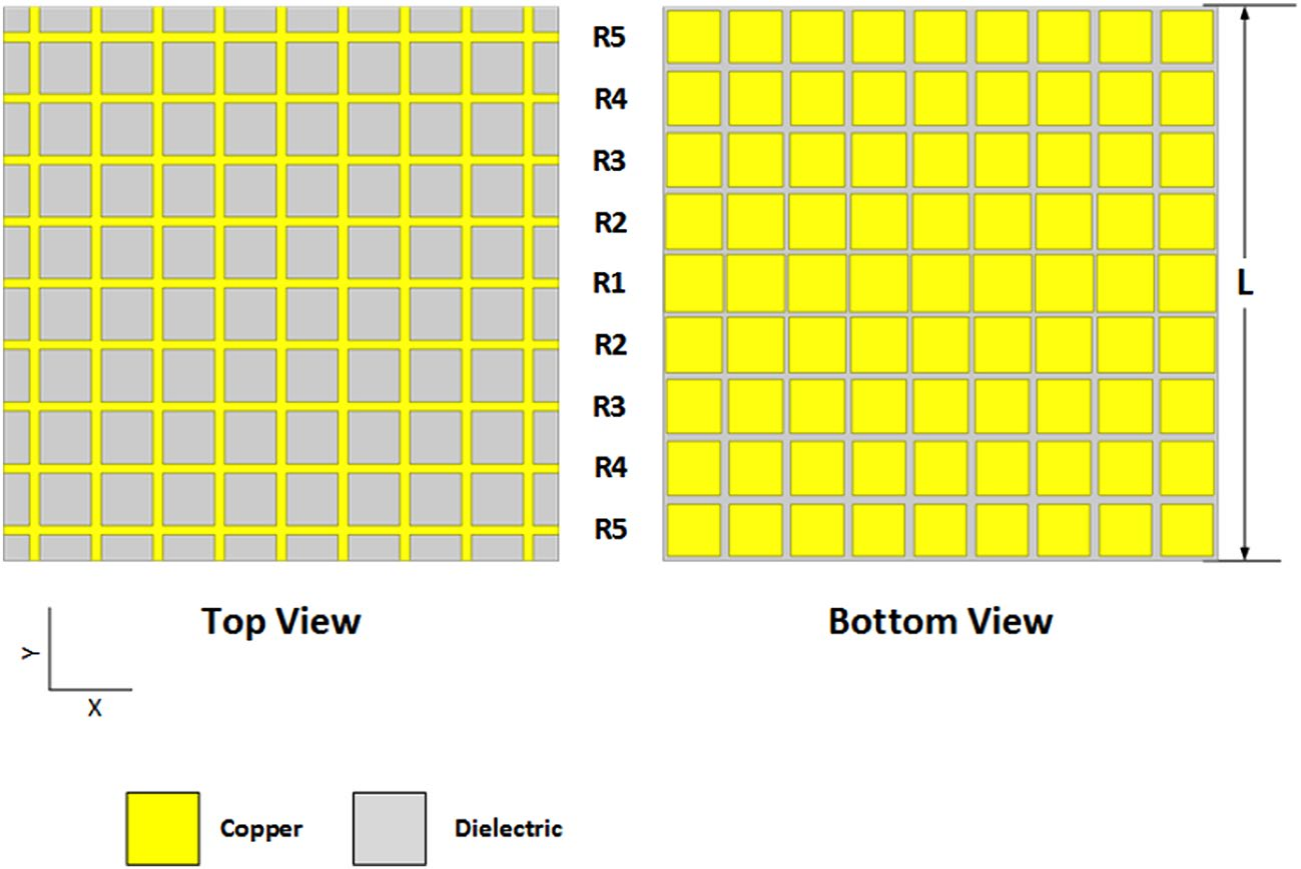
Top and bottom view of designed PRS.

Maximum beam tilt in an FP cavity-resonator-based antenna is achieved when the medium between the source and the PRS is air. For large values of beam tilts, large lateral size of the FP resonator is also required. A large beam tilt not only requires a large cavity size, but also a large lateral size of the FP cavity. Also, the side lobe levels (SLLs) increase at high tilt angles and causes an interfere with the radiation patterns of neighboring elements, which in turn increases the correlation. Taking all mentioned parameters into consideration, a PRS whose lateral size is L×L = 117 mm × 117 mm is designed with 9×9 unit cells, using design parameters mentioned above. The dimension of the PRS is kept small for the design to be more practical. Figure 3 shows the schematic diagram of the designed PRS. Row R1 is comprised of unit cells having capacitive patches whose side dimension is 12.2 mm, while the size of the remaining rows is reduced gradually until they reach 11.0 mm. The phase distribution against all these dimensions from 12.2 to 11.0 mm are mentioned in Fig. 2.

The *R*1 row, which has the maximum reflection coefficient is placed between element 1 (E1) and element 2 (E2). As we go towards both edges of the PRS (along y-axis) from this center, the reflection coefficient decreases progressively with each new row offering a lower reflection coefficient to the radiated waves. It should be noted that the transition among *R*1, *R*2 and *R*3 is deliberately kept sharper than the variation between *R*3, *R*4 and *R*5. This is done to ensure that the phases of all rows, especially the last row, remain negative. Also, a sharp variation is only required in the rows above the radiation sources. Eq. (2) predicts the antenna height by considering a uniform PRS without any phase gradient, so the height of the phase gradient PRS is not predicted exactly by this relationship. The phase gradient PRS height is mostly affected by the unit cells present directly above the radiating sources, which in our case are unit cell rows *R*1, *R*2 and *R*3.

### Complete system

**Figure 4:**
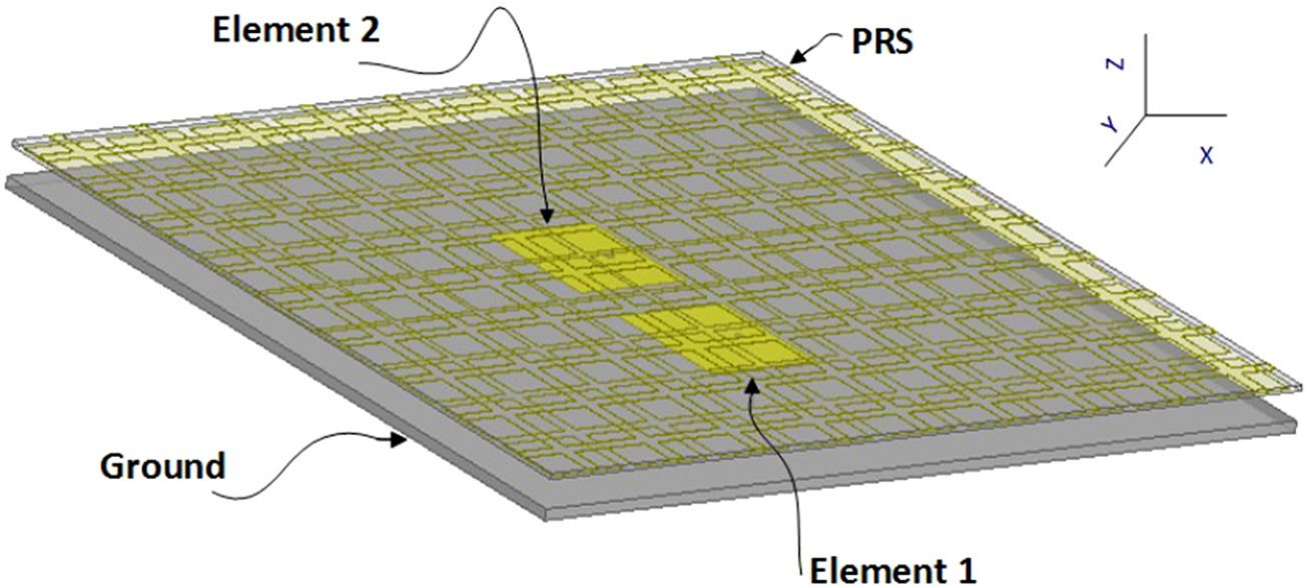
Proposed MIMO antenna design with PRS placed at *λ*/10 height.

To validate the performance of the designed PRS, two element (E1 and E2) MIMO patch antennas are designed to radiate at 5.36 GHz. The elements are printed on a Rogers DiClad 880 substrate with *ε*_*r*_ = 2.2 and a thickness of 1.578 mm. The length and width of each patch is 21.25 mm and 17.2 mm, respectively. The patches are excited using probe-feed at 5.1 mm offset to ensure impedance matching. The edge-to-edge distance between the patches is 8 mm (*λ*/7). In order to place the PRS above the antennas, the exact height cannot be predicted using Eq. (2) since the unit cells of the PRS are not uniform. Thus, the exact reflection phase is not known. However, it is understood that it lies between a value corresponding to the values of R1 and R5. A parametric study is carried out to determine the optimal height of the PRS above the radiating sources to make an FP cavity resonator. The optimum height is found to be 5.6 mm (*λ*/10) with its capacitive grid facing the MIMO antenna elements. The final design of the proposed MIMO antenna is depicted in Fig. 4.

## Results and discussion

After careful analysis of the design via simulations, a prototype is fabricated and measured. The performance is compared before and after placing the PRS with the main focus on the improvement in *ρ*. The following sub-sections discuss these results.

### Simulations results

**Figure 5:**
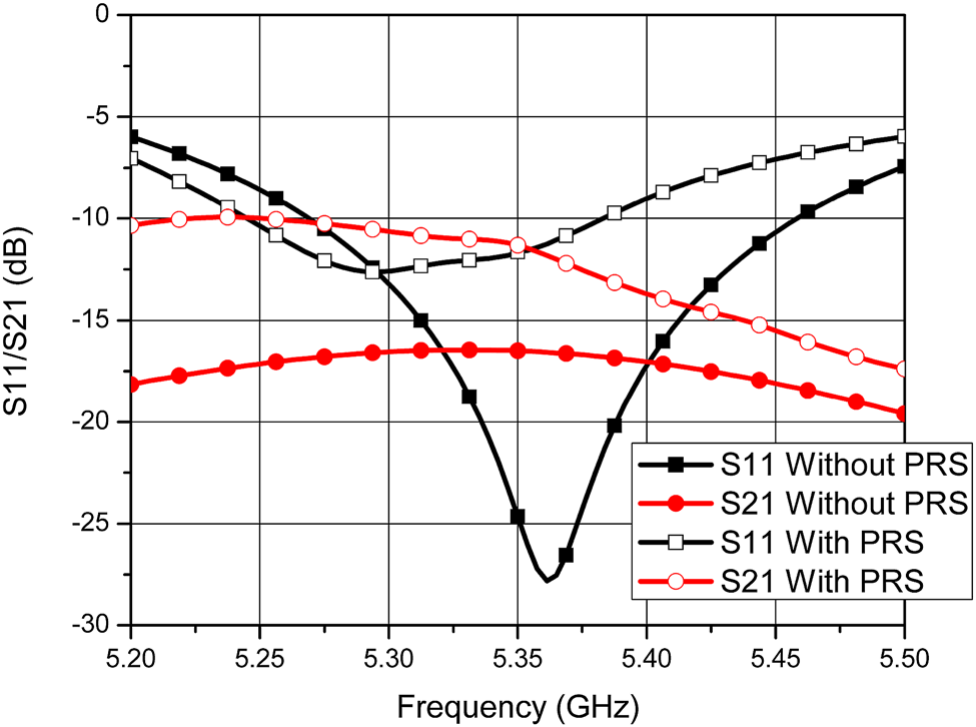
Simulated S-parameters of MIMO antenna with and without the PRS.

The S-parameters of the designed MIMO antenna are analyzed in the simulation software. Without the PRS, the antenna has a resonant frequency of 5.36 GHz with a 10 dB impedance bandwidth of 187.5 MHz. The minimum isolation between the antenna elements E1 and E2 is 16.8 dB. Once the PRS is placed over the antenna elements E1 and E2, the return loss degrades and the resonant frequency shifts to 5.32 GHz with a 10 dB bandwidth of 137.5 MHz. Due to the PRS placement, isolation between the antenna elements also degrades. However, it is an important design consideration to have a PRS with such unit cells and height above the radiating elements with port isolation greater than 10 dB. With the PRS placed at a height of 5.6 mm, the isolation between the antenna elements remains above 10 dB in the entire operating band of the MIMO antenna. Figure 5 shows the S-parameters of the proposed antenna with and without the placement of the PRS.

**Figure 6:**
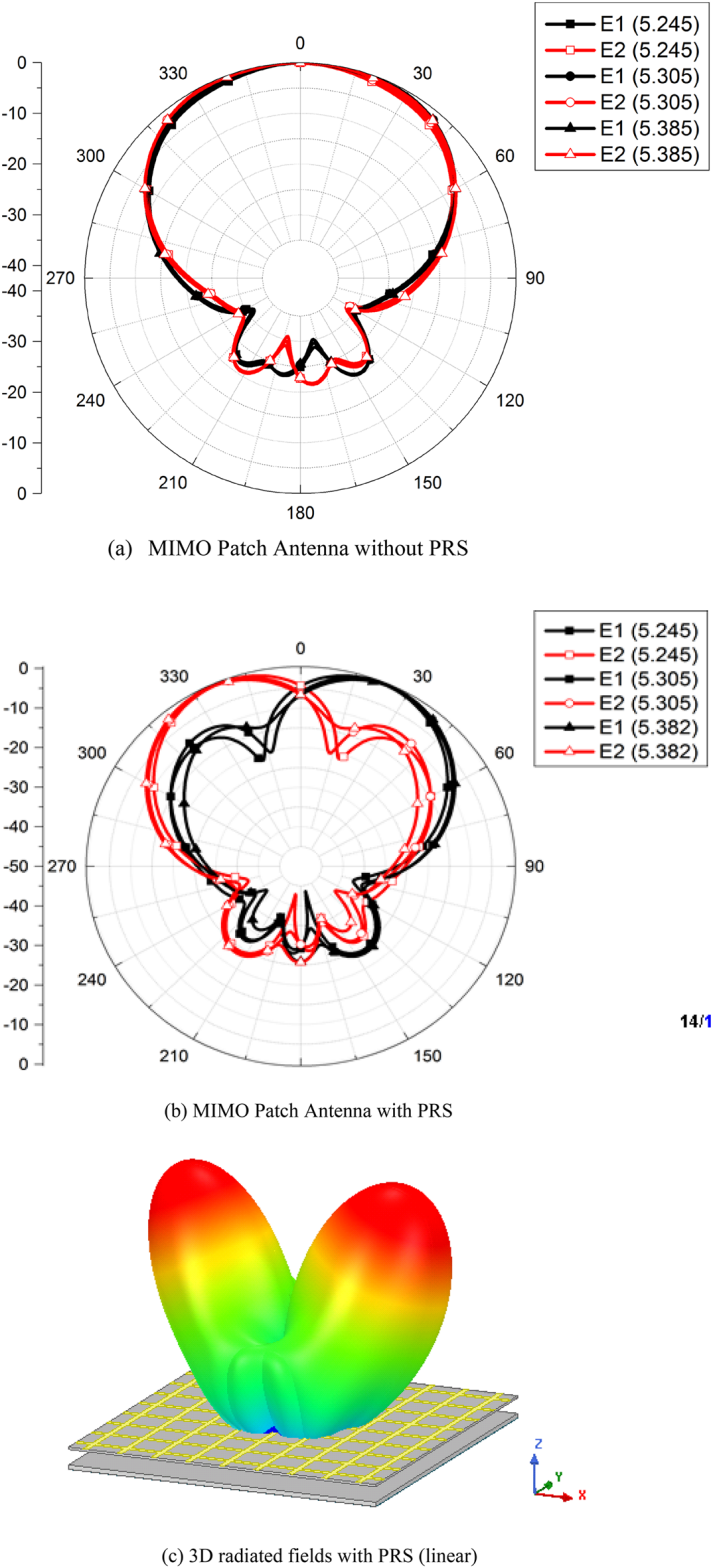
Radiation Pattern.

The far-field parameters are also analyzed from the simulations. The radiation patterns of the antenna without the PRS are calculated at multiple frequencies in the operating band of the antenna and shown in Fig. 6a. A high correlation between radiation patterns of the antenna elements E1 and E2 can be noticed from this figure which yields high *ρ*. Figure. 6b–c shows the radiation patterns of E1 and E2 once the PRS is placed over them. The patterns of the elements E1 and E2 are tilted away from each other due to the placement of PRS. Tilt angles for E1 and E2 are +24*°* and −24*°* degree, respectively, resulting in total difference of 48*°* at the frequency of interest.

**Figure 7:**
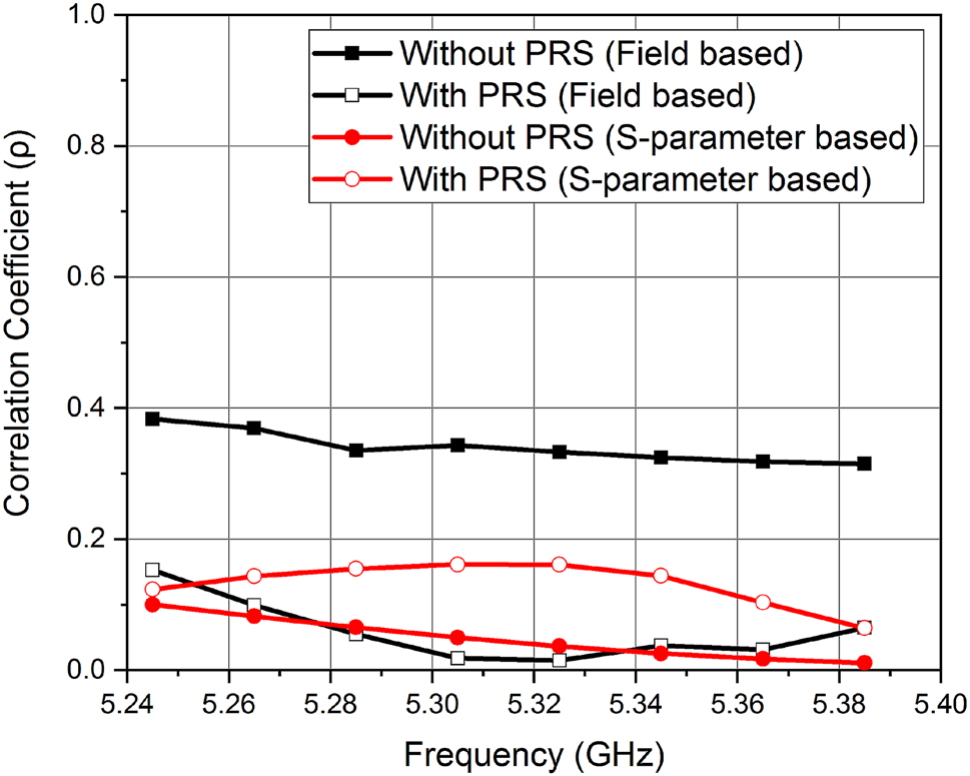
Calculated *ρ* Value using field-based equation and S-parameter-based equation.

Figure 7 summarizes the results of *ρ* calculated both from S-parameter-based equation presented in^2^ and from radiation patterns using Eq. (1), for both cases. It can be seen that, in the case with PRS, *ρ* is reduced significantly once calculated from the radiated fields. This is because the actual fields are now decorrelated. On the other hand, the S-parameter-based equation incorrectly shows the increase in *ρ* due to decrease in the port isolation. These results agree with the findings of^9^ that using the S-parameter-based equation for calculation of *ρ* often leads to inaccurate results.

### Measured results

**Figure 8:**
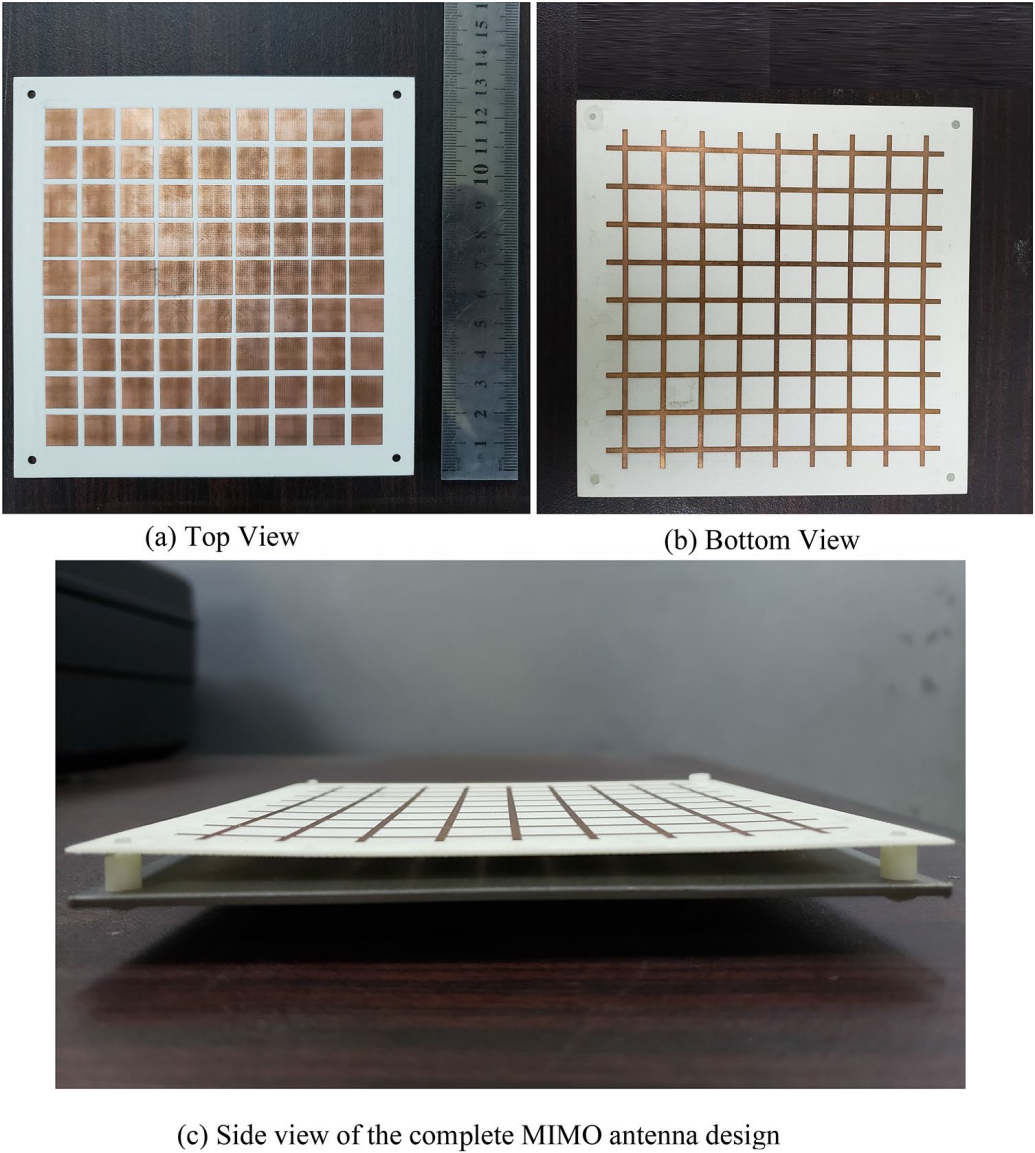
Fabricated Prototype.

The fabricated prototype of the proposed design is excited through two coax connectors placed on the bottom of radiating sources. Design is measured for S-parameters and the far-field parameters to verify its operation. Figure 8 shows the fabricated PRS and the complete MIMO antenna design. To place the PRS over the antenna, four spacers of height 5.6 mm are placed around its corners. Plastic spacers are used to ensure that they do not interfere with the e pattern of the antenna.

**Figure 9:**
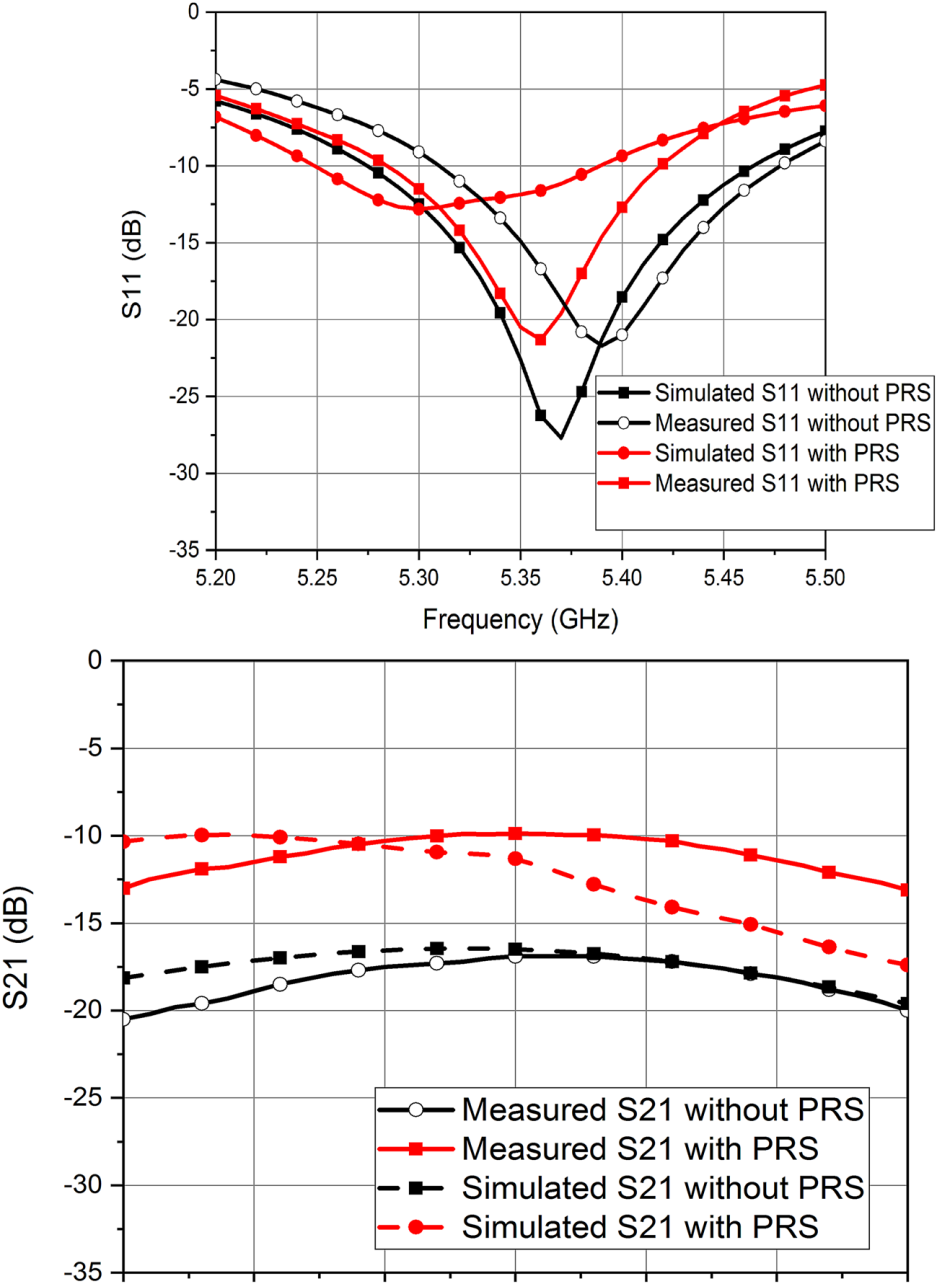
Measured vs Simulated Results.

The S-parameter measurements of the fabricated design are performed without and with the PRS placed over the MIMO antenna. The MIMO antenna has a resonant frequency of 5.39 GHz without the PRS which is slightly shifted towards the higher side as compared to the simulations. This is mainly due to the dielectric constant tolerance of the substrate on which the antenna is designed. The measured bandwidth of each patch antenna element is 160 MHz. Once the PRS is placed, the resonant frequency of the antenna shifts to 5.36 GHz with a bandwidth of 130 MHz, which is in agreement with the simulation results. Figure 9 compares the simulated and measured S-parameters of the designed antenna and shows a good agreement between the two.

**Figure 10:**
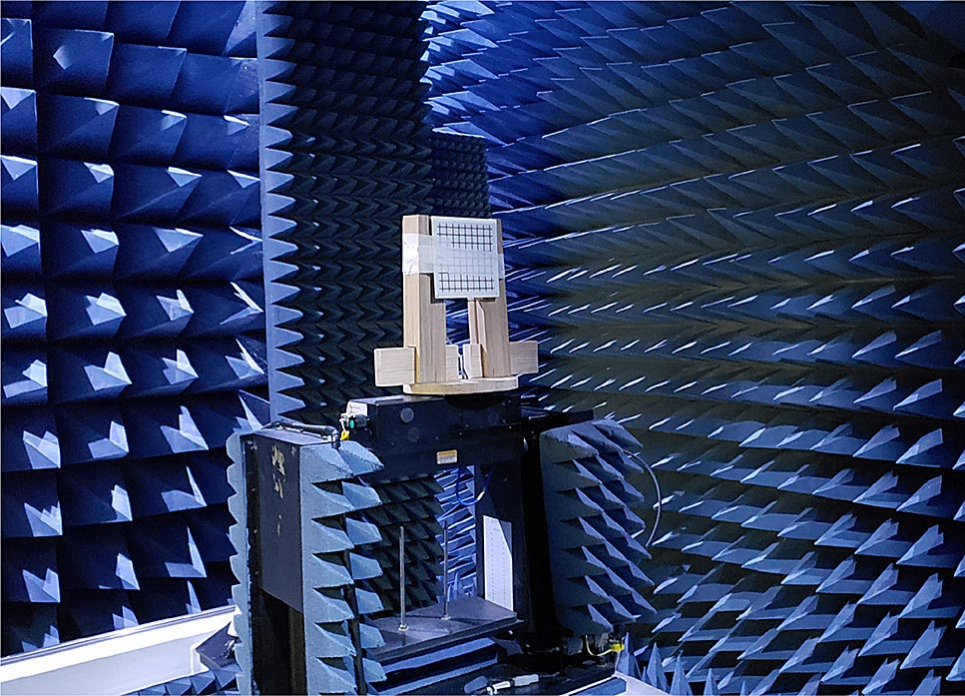
Measurement Setup.

**Figure 11:**
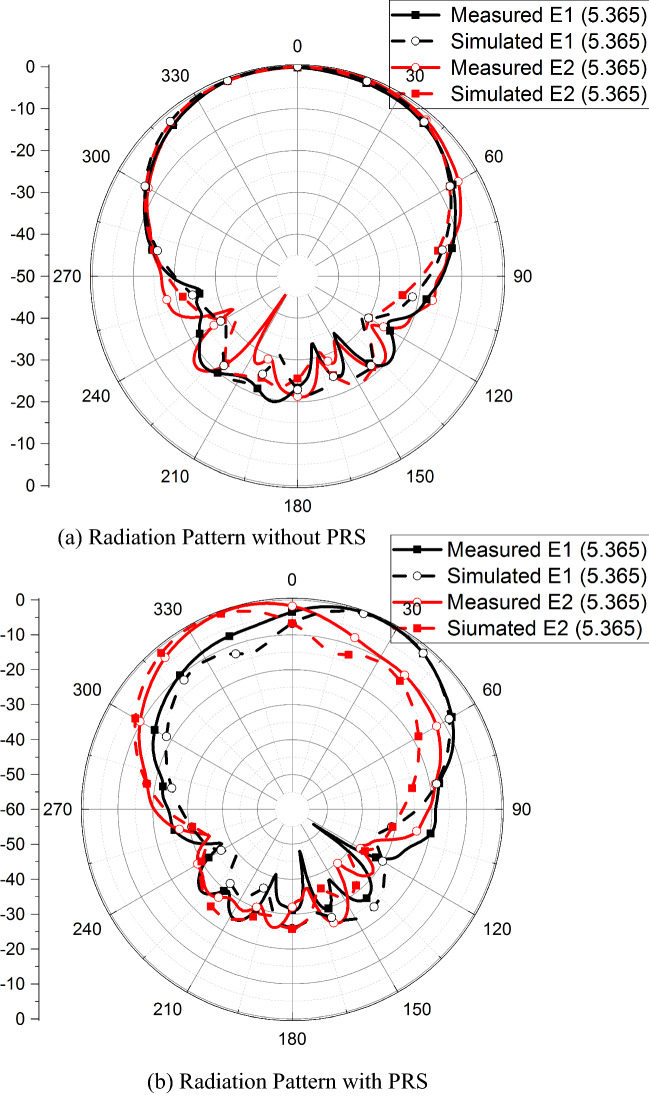
Measured vs Simulated Results.

Radiation patterns and gain measurements of the fabricated prototype are performed in a far-field anechoic chamber. Figure 10 shows the measurement setup of proposed antenna in anechoic chamber. While conducting measurements of one antenna element, the port of the other antenna element is terminated with a 50 Ω matched load. Gain of the antenna without the PRS is measured to be 6.21 dB at the resonant frequency for both the antenna elements E1 and E2. With the PRS placed over the antenna elements, the gain increases to 9.12 dB indicating an increase of 2.91 dB. This is mainly due to the beams of each antenna element getting narrower in the FP cavity configuration. Figure 11 shows the simulated and measured radiation patterns of the antenna without and with the PRS at the resonant frequency. The beam of the antenna element E1 tilts to +24*°* while that of E2 tilts to −24*°* as predicted by the simulation results, thus decreasing the *ρ*.

Finally, a comparison between the design presented by^10^ and this work is shown in Table 2. The beam tilt for^10^ was reported to be 31^0^, while it is 24^0^ in our design, and the reduction in *ρ* is comparable. The reason being that the reported directivity of^10^ was 10 dB, while in this work it is 11.92 dB at the resonant frequency. An increase in the beam directivity leads to less overlap of the radiation pattern and, hence, a decrease in the correlation coefficient. Another important parameter is the isolation between the antenna elements E1 and E2. In^10^ the isolation is 11.92 dB with a cavity height of *λ* /4, while in this work, even with a reduced cavity height of *λ*/10, minimum isolation of 10 dB is ensured in the entire operating band of the antenna.

**Table 2:** Proposed designs of low-profile FP in literature for single source element.

Ref	% BW	Dimension	s Beam tilt	Unit Cell	*ρ * Re-duction
^10^	2.6%	1.84*λ* × 1.84*λ* × *λ* /4	31^0^	Square capacitive patch and inductive apertures	99%
This work	3.5%	2.07*λ* × 2.07*λ* × *λ* /10	24^0^	Square capacitive patch and inductive grid	95%

## Conclusion

In this paper, the design of a low profile FP cavity-based MIMO patch antenna with reduced correlation coefficient is presented for compact communication devices such as wireless access points. Reduction in *ρ* is achieved by decorrelating the radiated fields of individual antenna elements. Lateral size of the structure is also kept small for practical reasons. This *ρ* reduction is achieved with closely placed elements (*λ*/7) while maintaining acceptable coupling. The height of the FP cavity achieved is *λ*/10 at 5.36 GHz which is much lower than previously reported *λ*/4. For this height, a decrease of 95% in *ρ* is achieved when compared to a simple MIMO patch antenna. Although the height of the proposed design can be reduced even further, this may cause increased coupling between the antenna elements and, hence, may not lead to any significant decrease in *ρ*.

## Data Availability

All data required to evaluate the findings of this work is available in the presented paper. Additional data related to this work may be requested from the corresponding author.
